# Identification of a Novel Cetacean Polyomavirus from a Common Dolphin (*Delphinus delphis*) with Tracheobronchitis

**DOI:** 10.1371/journal.pone.0068239

**Published:** 2013-07-10

**Authors:** Simon J. Anthony, Judy A. St. Leger, Isamara Navarrete-Macias, Erica Nilson, Maria Sanchez-Leon, Eliza Liang, Tracie Seimon, Komal Jain, William Karesh, Peter Daszak, Thomas Briese, W. Ian Lipkin

**Affiliations:** 1 Center for Infection and Immunity, Mailman School of Public Health, Columbia University, New York, New York, United States of America; 2 EcoHealth Alliance, New York, New York, United States of America; 3 Department of Pathology and Research, SeaWorld Parks, San Diego, California, United States of America; 4 Wildlife Conservation Society, Bronx Zoo, New York, New York, United States of America; NIAID, United States of America

## Abstract

A female short-beaked common dolphin calf was found stranded in San Diego, California in October 2010, presenting with multifocal ulcerative lesions in the trachea and bronchi. Viral particles suggestive of polyomavirus were detected by EM, and subsequently confirmed by PCR and sequencing. Full genome sequencing (Ion Torrent) revealed a circular dsDNA genome of 5,159 bp that was shown to form a distinct lineage within the genus *Polyomavirus* based on phylogenetic analysis of the early and late transcriptomes. Viral infection and distribution in laryngeal mucosa was characterised using in-situ hybridisation, and apoptosis observed in the virus-infected region. These results demonstrate that polyomaviruses can be associated with respiratory disease in cetaceans, and expand our knowledge of their diversity and clinical significance in marine mammals.

## Introduction

Polyomaviruses (PyVs; family: *Polyomaviridae*) are small non-enveloped viruses with icosahedral capsids of 40–45 nm and a circular dsDNA genome of approximately 5 kbp [1]–[3]. They exhibit bidirectional transcription, which begins early in the infectious cycle with the non-structural large and small tumour (T) antigens (T-Ag/t-Ag). These proteins are produced following alternative splicing of a common pre-messenger RNA (mRNA), and serve multifunctional roles in the regulation of viral and host gene expression and viral DNA replication. In mammalian polyomaviruses, these proteins can also induce and maintain neoplastic transformation in cell culture, or neoplasias *in vivo* [4]. The structural proteins VP1, VP2 and VP3 form the icosahedral viral capsid, and facilitate cell entry. Coding mRNAs for these proteins are transcribed later in the infection, initiated by the early T antigens, and like the early proteins are also produced from overlapping coding sequences that are alternatively spliced from a common mRNA. In several members of the mammalian polyomaviruses an additional ‘agnoprotein’ is also expressed, which is thought to be associated with capsid assembly and enhancing viral release [5].

Polyomaviruses infect a wide range of avian and mammalian hosts with varying clinical significance. In avian hosts infection is often associated with acute and severe disease, while mammalian PyVs generally result in mild or subclinical infections [2] unless the host is immunosuppressed [6]–[9]. Significantly, our knowledge of PyV epidemiology and pathogenesis is mostly limited to terrestrial species, and the only reported marine PyV infection is in a California sea lion (*Zalophus californianus*) with small proliferative lesions on the dorsal mucosa of the tongue [10], [11]. Here we describe a novel cetacean PyV and implicate the virus in the death of a free-ranging short-beaked common dolphin (*Delphinus delphis*).

## Results and Discussion

**Figure 1:**
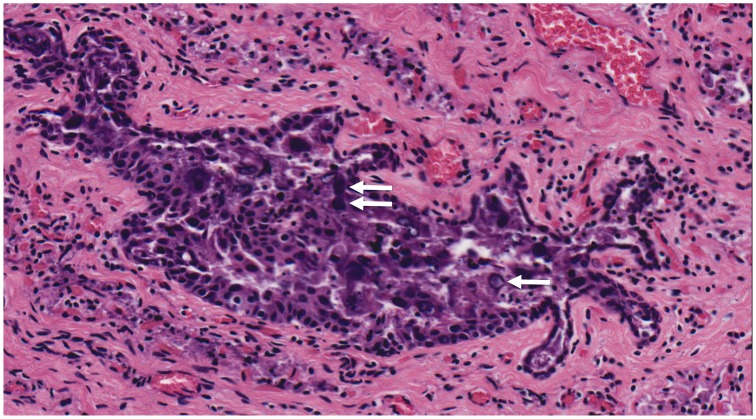
There is multifocal attenuation and necrosis of the epithelium and filling of the lumen with debris and sloughed epithelial cells. Multifocal sloughed cells demonstrate karyomegaly with basophilic intranuclear inclusions (arrows). HE stain at 20× magnification.

A female common dolphin calf was found stranded in San Diego, California in October 2010, but died before rescue could be attempted. Gross and histologic examination revealed a moderate to severe diffuse hepatic lipidosis associated with an acute anorexia. In the upper airways, multifocal ulcerative lesions were observed in the trachea and bronchi with epithelial loss, and haemorrhage with necrotic and inflammatory debris admixed with sloughed epithelial cells within the airway lumens. Occasional cells in the laryngeal mucosa demonstrated karyomegaly with large, pale basophilic intranuclear inclusions, suggesting a viral aetiology to the tracheobronchitis (Figure 1). Malaise from the airway infection likely caused the anorexia and the subsequent hepatic lipidosis. No changes suggestive of viral inclusions were noted in other organs.

Electron microscopy (EM) of laryngeal mucosa revealed loosely dispersed intranuclear virus particles of 45–55 nm with structural morphology consistent with viruses from the families *Polyomaviridae* and *Papillomaviridae* [3]. PCR analysis of nucleic acids extracted from sections of formalin-fixed paraffin-embedded (FFPE) laryngeal mucosa revealed polyomavirus VP1 (∼250 bp) and VP3 (∼400 bp) sequences, and quantitative PCR (qPCR) targeting VP1 indicated 2.5×10^6^ genome copies per 100 ng of DNA. Identical sequences were also obtained from the lung, though no pathologic changes were noted here on histologic review. Viral load in the lung was 8.0×10^2^ genome copies per 100 ng, which is approximately three orders of magnitude less than the trachea, suggesting that virus is probably shedding into the lung from ulcerative foci on the adjacent bronchi. Frozen liver, spleen, brain and serum were also screened for viral load but no PyV was detected. Consensus PCR assays for other viruses known to infect marine mammals including: papillomaviruses, herpesviruses, paramyxoviruses, poxviruses, adenoviruses and caliciviruses, were also negative for all samples.

**Figure 2:**
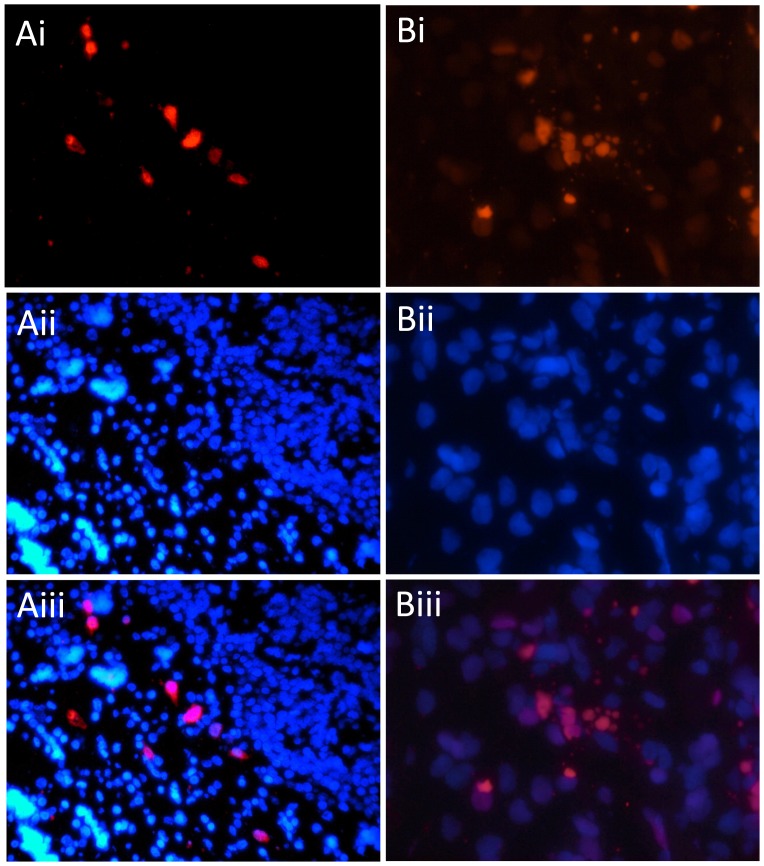
Infection of dolphin trachea with DPyV-1 and demonstration of polyomavirus ISH staining and apoptosis in virus infected tissue. A = ISH and DAPI infected trachea at ×40 magnification. Panel Ai = DPyV-1 ISH staining (red) showing intranuclear infection of mucosal tissue. Panel Aii = DAPI staining for cell nuclei. Aiii = Merge of ISH and DAPI. B = TUNEL-TMR staining (red) for apoptosis at ×100 magnification. Panel Bi = TUNEL staining of apoptotic cell nuclei. Bii = DAPI staining. Biii = Merge of TUNEL and DAPI. Tracheal mucosa of an uninfected common dolphin was used as a negative control and no staining for virus or apoptosis was observed (data not shown).

In-situ hybridization (ISH; [12]), with oligonucleotide probes targeted to the partial VP1 and VP3 sequences confirmed PyV infection in the laryngeal mucosa (Figure 2). Cellular morphology of infected cells and terminal deoxynucleotidyl transferase dUTP nick end labeling (TUNEL) consistent with apoptosis was also observed in the virus-infected region (Figure 2), but not in laryngeal mucosa of uninfected control tissue. Because TUNEL staining cannot distinguish whether the apoptosis in infected tissue was virally or host-mediated, either should be considered in the interpretation of these results, as shown previously [13], [14]. While our data do not prove causation, the localisation of this virus with pathology supports a clinical association between the PyV and the observed tracheobronchitis.

Ion Torrent deep sequencing of total nucleic acid extracted from the FFPE laryngeal tissue yielded a total of 924,665 reads after ambiguous nucleotides, primers or adaptor sequences were removed. These were pooled to enable assembly of a full genome comprising 5,159 bp at ∼20× coverage. The full sequence was confirmed by PCR amplification and classical dideoxy sequencing of 500 bp overlapping fragments across the genome (GenBank accession no. KC594077). Identical full-length sequence was also obtained from the lung material. Given the host in which this virus was discovered, we propose that it be named *Dolphin polyomavirus 1* (DPyV-1).

**Figure 3:**
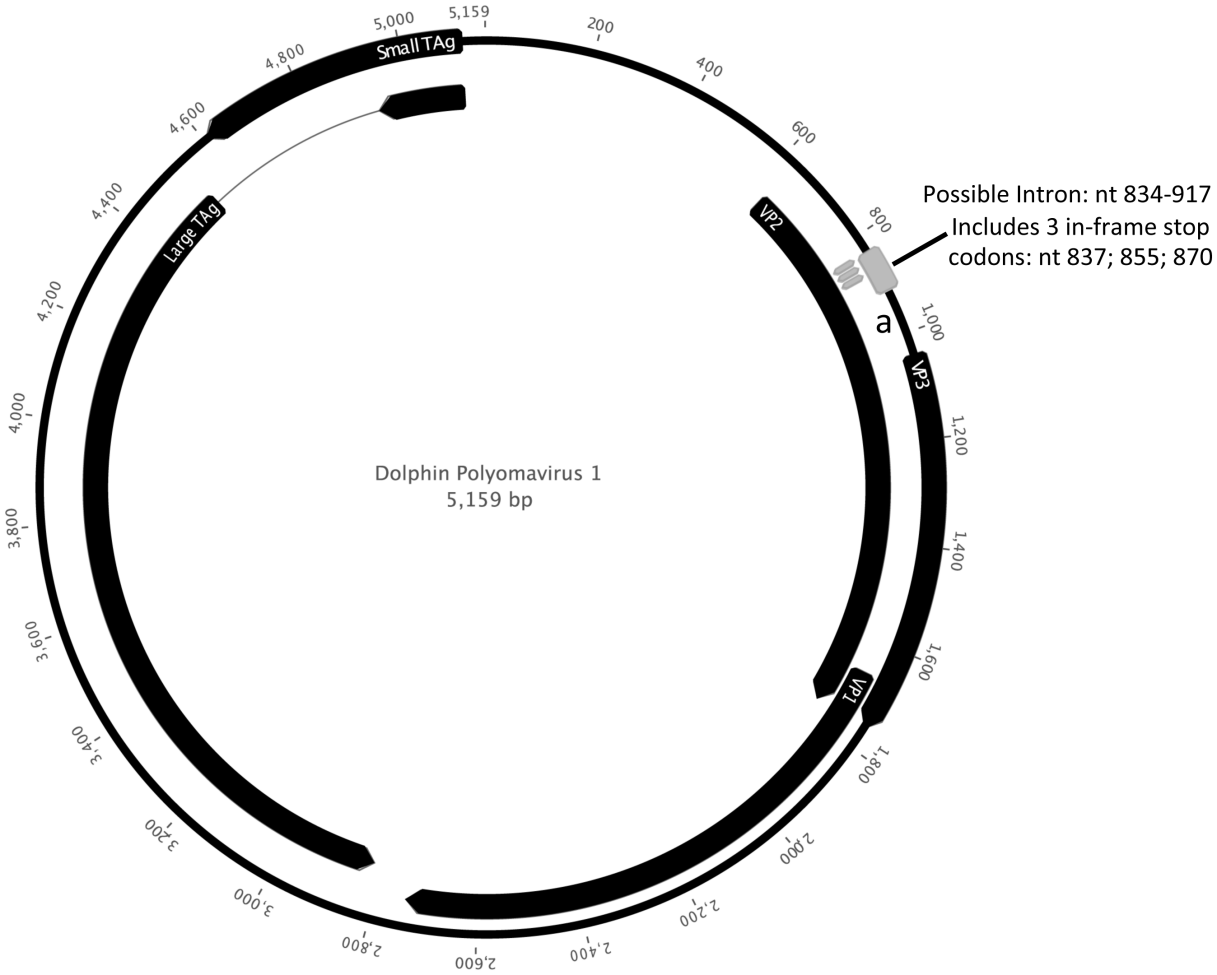
Genome arrangement of DPyV-1 (accession number: KC594077). Possible intron in VP2 indicated, containing three in-frame stop codons. Truncation of the VP2 by any of these stop codons considered unlikely as the region downstream of the intron (a) shows strong homology to VP2.

Coding sequences of the early (large and small T-antigens) and late (VP1, VP2 and VP3) genes were identified based on genomic location and on homology to known polyomavirus proteins. The genome arrangement is presented in Figure 3. Analysis of the late genes revealed two regions with no apparent homology to any known PyV. First, the VP2 ORF includes a region (nucleotides 814–892) without homology to other VP2 sequences that contains three in-frame stop codons at positions 837–839, 855–857 and 870–872, which would lead to a truncated VP2 protein since translational read-through at three consecutive stop sites appears very unlikely. However, regained sequence homology with other VP2 sequences just downstream of the last stop codon, even in the sequence portion not overlapping with VP3 (nucleotides 893–1043), could be compatible with that sequence representing a potential intron. In support of this hypothesis, sequences resembling classical eukaryotic splice consensus signals are discernable flanking the divergent region (CAG_813_/GUaGg – CUu[A]g – TTTTTaCAG/G_892_). The second region is an apparent insertion further downstream in the VP2/VP3 open reading frame (nucleotides 1305–1382), which does not show obvious homology to sequenced polyomavirus VP2/3s. The significance of these genetic features remains to be determined.

**Figure 4:**
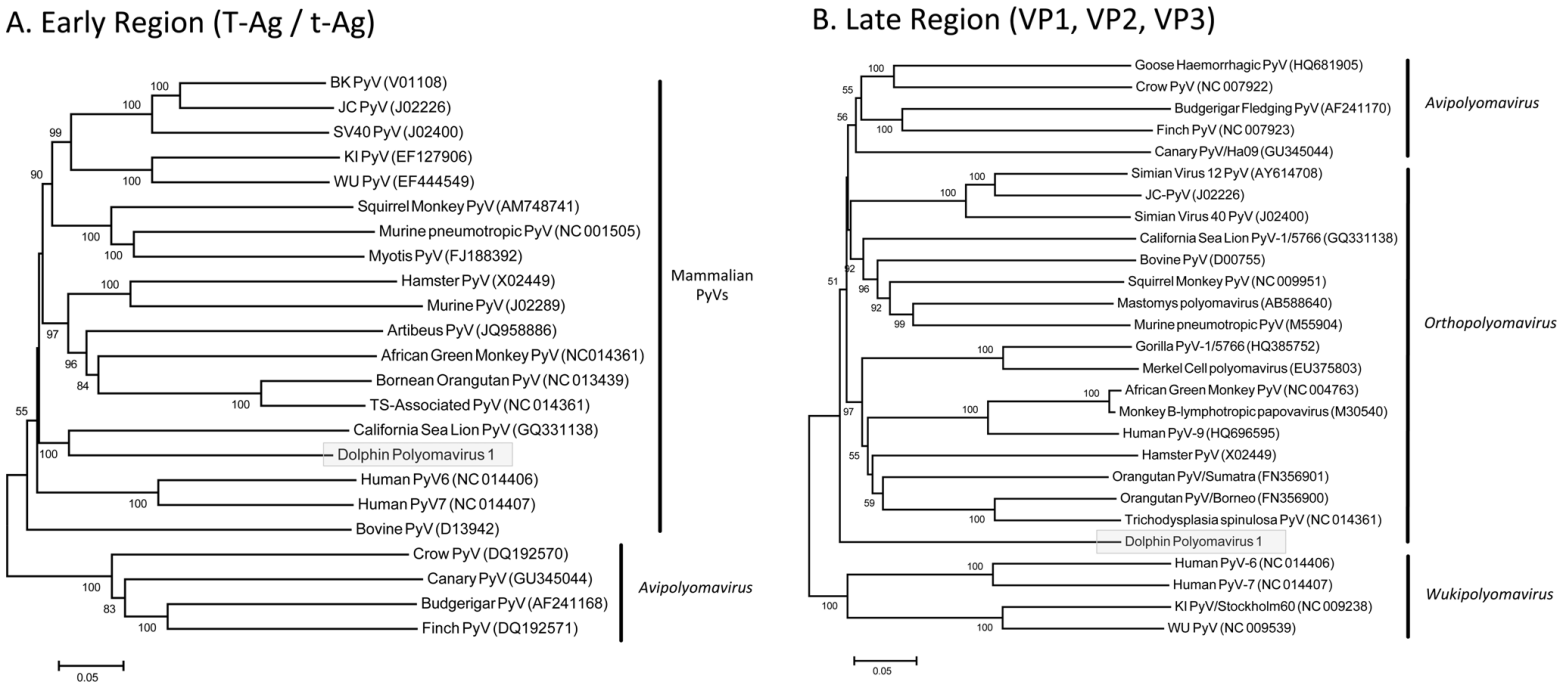
Phylogenetic trees based on complete A) early (T Antigen) and B) late (VP1, VP2 and VP3) transcripts. Alignments were generated using Muscle (executed in Geneious V6.0.4), and Maximum Likelihood trees generated in Mega V.5.

Phylogenetic analysis of the late region (VP1, VP2 and VP3) produced trees in agreement with the proposed taxonomic revisions for the family *Polyomaviridae* [1]; however deep nodes were generally poorly supported with low bootstrap values (Figure 4). DPyV-1 did not cluster with bovine or sea lion PyVs, despite previous suggestions that viruses from laurasiatherian hosts (bats, carnivores, ungulates and cetaceans) form a monophyletic clade [11]. This observation suggests either that host selection is not reflected in the late transcriptome, or that DPyV-1 has a distinct evolutionary history (such as spillover from another host). These results suggest that DPyV-1 belongs to a hitherto un-described lineage within the family *Polyomaviridae,* which may reflect a marine origin. Analysis of the early region (T antigens) also produced trees in agreement with previous analyses [1], [11]. Based on this region, DPyV-1 is most closely related to the California sea lion virus (Figure 4), however the phylogenetic relatedness of PyVs was generally more ambiguous in this region and no separation of the proposed mammalian genera was discernable; thus the significance of this placement is unknown.

Next steps in this investigation will include efforts to determine the prevalence of DPyV-1 in different populations of short-beaked common dolphins to better understand the significance of this virus to dolphin morbidity and mortality. To our knowledge, there are currently no other reports of similar respiratory concerns in delphinids with changes suggestive of viral inclusions or associated with PyV. A review of the records of one of the authors (JS) demonstrated just over 500 cetaceans with good tissues for respiratory tract evaluation. In none of these cases was this viral infection suspected. However, PCR EM and PCR evaluations are not routine so the true incidence is unknown at this time. As the field of marine mammal virology expands, we expect that more cases will be detected based on a heightened index of suspicion. Respiratory disease is a common concern in cetaceans, and while the main etiologic concerns are bacterial and fungal, a primary viral condition is often not evaluated. Our results do however suggest that PyVs have the capacity to cause respiratory disease in cetaceans, and further contribute to comparisons of viral diversity between terrestrial and marine ecosystems.

## Materials and Methods

### Sample Collection

Tissue samples were collected post-mortem from a female juvenile dolphin, and preserved for medical diagnostics in either formalin, or directly frozen.

### Extractions and PCR

Nucleic acids were extracted from 5×8 um sections of FFPE tissues using the RecoverAll™ Total Nucleic Acid kit (Ambion®, Catalogue #AM1975), according to the manufacturer’s instructions. Nucleic acids were extracted from fresh frozen tissues using the MagNA Pure 96 Purification System (Roche), according to the manufacturer’s instructions. Consensus PCR using broadly reactive primers were used for the detection of polyomavirus VP1 and VP3 [15], papillomaviruses, [16], herpesviruses [17], paramyxoviruses [18], poxviruses [19], adenoviruses [20] and caliciviruses [21]. Synthetic RNA/DNA constructs (targeting a representative virus for each family) were used as positive controls for all PCR assays, and all were successfully amplified. Viral load of PyV was assessed using quantitative (q) Real-Time PCR, with primers and probe targeting 158 bp of the VP1 fragment. Primer sequences were VP1/qPCR/FWD: GATGCCAGTCATCATGCTTTCCTCA, VP1/qPCR/RVS: GCCCCCTGCATACTTTGCCCT and VP1/qPCR/Probe: FAM- GGCCTACCTAGATTCTTCAGAGTAGG-TAMRA. Standard curves were generated by cloning the VP1 fragment generated by the PyV consensus PCR using StrataClone PCR Cloning kits (Agilent Technologies), according to the manufacturer’s instructions. Plasmid DNA was purified using PureLink™ Quick Miniprep kit (Life Technologies), linearised, and log dilutions (10^6^–10^1^) prepared. Real-time PCR was performed using TaqMan® Universal PCR Master Mix, according to the manufacturer’s instructions.

### Ion Torrent Sequencing

Libraries for ion torrent deep sequencing were prepared following the Ion Xpress™ Plus gDNA Fragment Library Preparation protocol with minor modifications. Briefly, DNase treated RNA (FFPE-extracted laryngeal tissue), was subjected to random first strand synthesis using SuperScript III in the presence of RNase out, according to the manufacturer’s instructions. cDNA and RNase treated DNA were incubated with 20 U of Klenow DNA polymerase at 37°C for 45 minutes. Enzymatic fragmentation was performed by incubating Agencourt® AMPure® XP purified double stranded cDNA with 10 µL of Ion Shear™ Plus Enzyme Mix II and 5 µL of Ion Shear™ Plus 10X Reaction Buffer at 37°C for 15 min. Purified fragmented DNA was barcode and adapter ligated by mixing it with 10 µL of 10X ligase buffer, 2 µL of Ion P1 adapter, 2 µL of Ion Xpress Barcode, 2 µL of dNTP mix, 49 µL of nuclease-free water, 2 µL of DNA ligase and 8 µL of nick repair polymerase. The mixture was incubated at 25°C for 15 min and 72°C for 5 min. PCR of ligated and purified DNA product was performed by using 25 µL of ligated product, 100 µL of Platinum PCR Super Mix High Fidelity and 5 µL of library amplicon primer. Temperature cycling conditions were: 1 cycle of 95°C-5 min and 8 cycles of 95°C-15 sec, 58°C-15 sec, 70°C-1 min. Amplified DNA was subjected to Agencourt® AMPure® XP purification. Quantification and qualification of the barcoded libraries was performed on the Bioanalyzer with Agilent High Sensitivity DNA kit and 150 bp average size libraries were used for sequencing template preparation.

Template preparation for sequencing was performed by following the standard Ion One Touch™ 200 Template protocol. Briefly 20 µL of diluted library pool was mixed with 280 µL of nuclease-free water, 500 µL of Ion One Touch 2× Reagent Mix, 100 µL of Ion OneTouch Enzyme mix and 100 µL of Ion OneTouch 200 Ion Sphere Particles for performing clonal amplification. Recovered template-positive Ion sphere particles (ISPs) were subjected to enrichment according to template corresponding protocol. Ion Sphere quality control protocol was performed on enriched and unenriched template ISPs. Samples containing sufficient number of template ISPs and satisfactory enrichment were subjected to the standard Ion PGM™ 200 Sequencing protocol.

### Sequence Analysis

Ion Torrent raw reads were imported into Geneious (V.6.0.4) and 18 nucleotides trimmed from the 5′ and 3′ ends. The VP1 and VP3 sequences obtained through consensus PCR were then used a seed for the assembly of full length PyV sequence, also executed in Geneious. Nucleotide sequences alignments were made using using ClustalW, executed in Geneious (V6.0.4). Phylogenetic trees were constructed using Neighbour-Joining and Maximum Likelihood algorithms in Mega (V.5) and bootstrapped using 1000 replicates.

### Molecular Pathology

Fluorescent in-situ hybridization (FISH) was performed using the Quantigene View RNA ISH Tissue Assay (Affymetrix), according to the manufacturer’s instructions, and as described previously [12]. FISH conditions were optimised to include a 10 min boiling and 20 min protease treatment. Oligonucleotide probes were designed commercially by Affymetrix using sequences of the polyomavirus VP1 and VP3. TUNEL staining was performed using the TMR-*In Situ* Cell Death Detection Kit (Roche) with deparaffinisation and protease treatment as described for the FISH protocol.

## References

[1] Johne R, Buck CB, Allander T, Atwood WJ, Garcea RL, et al (2011) Taxonomical developments in the family Polyomaviridae. Arch Virol 156: 1627–1634. 21562881 10.1007/s00705-011-1008-x PMC3815707

[2] Krumbholz A, Bininda-Emonds OR, Wutzler P, Zell R (2009) Phylogenetics, evolution, and medical importance of polyomaviruses. Infect Genet Evol 9: 784–799. 19379840 10.1016/j.meegid.2009.04.008

[3] King AMQ, Adams MJ, Carstens EB, Lefkowitz EJ, editors (2012) ICTV, Ninth Report of the International Committee on Taxonomy of Viruses.: Elsevier Academic Press.

[4] Ahuja D, Saenz-Robles MT, Pipas JM (2005) SV40 large T antigen targets multiple cellular pathways to elicit cellular transformation. Oncogene 24: 7729–7745. 16299533 10.1038/sj.onc.1209046

[5] Gerits N, Moens U (2012) Agnoprotein of mammalian polyomaviruses. Virology 432: 316–326. 22726243 10.1016/j.virol.2012.05.024 PMC7111918

[6] van der Meijden E, Janssens RW, Lauber C, Bouwes Bavinck JN, Gorbalenya AE, et al (2010) Discovery of a new human polyomavirus associated with trichodysplasia spinulosa in an immunocompromized patient. PLoS Pathog 6: e1001024. 20686659 10.1371/journal.ppat.1001024 PMC2912394

[7] Mourez T, Bergeron A, Ribaud P, Scieux C, de Latour RP, et al (2009) Polyomaviruses KI and WU in immunocompromised patients with respiratory disease. Emerg Infect Dis 15: 107–109. 19116066 10.3201/1501.080758 PMC2662633

[8] Cimbaluk D, Pitelka L, Kluskens L, Gattuso P (2009) Update on human polyomavirus BK nephropathy. Diagn Cytopathol 37: 773–779. 19626630 10.1002/dc.21147

[9] Ferenczy MW, Marshall LJ, Nelson CD, Atwood WJ, Nath A, et al (2012) Molecular biology, epidemiology, and pathogenesis of progressive multifocal leukoencephalopathy, the JC virus-induced demyelinating disease of the human brain. Clin Microbiol Rev 25: 471–506. 22763635 10.1128/CMR.05031-11 PMC3416490

[10] Colegrove KM, Wellehan JF Jr, Rivera R, Moore PF, Gulland FM, et al (2010) Polyomavirus infection in a free-ranging California sea lion (Zalophus californianus) with intestinal T-cell lymphoma. J Vet Diagn Invest 22: 628–632. 20622238 10.1177/104063871002200422

[11] Wellehan JF Jr, Rivera R, Archer LL, Benham C, Muller JK, et al (2011) Characterization of California sea lion polyomavirus 1: expansion of the known host range of the Polyomaviridae to Carnivora. Infect Genet Evol 11: 987–996. 21453794 10.1016/j.meegid.2011.03.010

[12] Anthony SJ, St Leger JA, Pugliares K, Ip HS, Chan JM, et al (2012) Emergence of fatal avian influenza in New England harbor seals. MBio 3: e00166–00112. 22851656 10.1128/mBio.00166-12 PMC3419516

[13] Andrabi S, Gjoerup OV, Kean JA, Roberts TM, Schaffhausen B (2007) Protein phosphatase 2A regulates life and death decisions via Akt in a context-dependent manner. Proc Natl Acad Sci U S A 104: 19011–19016. 18006659 10.1073/pnas.0706696104 PMC2141899

[14] van Gorder MA, Della Pelle P, Henson JW, Sachs DH, Cosimi AB, et al (1999) Cynomolgus polyoma virus infection: a new member of the polyoma virus family causes interstitial nephritis, ureteritis, and enteritis in immunosuppressed cynomolgus monkeys. Am J Pathol 154: 1273–1284. 10233865 10.1016/S0002-9440(10)65379-5 PMC1866551

[15] Johne R, Enderlein D, Nieper H, Muller H (2005) Novel polyomavirus detected in the feces of a chimpanzee by nested broad-spectrum PCR. J Virol 79: 3883–3887. 15731285 10.1128/JVI.79.6.3883-3887.2005 PMC1075742

[16] Forslund O, Antonsson A, Nordin P, Stenquist B, Hansson BG (1999) A broad range of human papillomavirus types detected with a general PCR method suitable for analysis of cutaneous tumours and normal skin. J Gen Virol 80 (Pt 9): 2437–2443. 10.1099/0022-1317-80-9-2437 10501499

[17] VanDevanter DR, Warrener P, Bennett L, Schultz ER, Coulter S, et al (1996) Detection and analysis of diverse herpesviral species by consensus primer PCR. J Clin Microbiol 34: 1666–1671. 8784566 10.1128/jcm.34.7.1666-1671.1996 PMC229091

[18] Tong S, Chern SW, Li Y, Pallansch MA, Anderson LJ (2008) Sensitive and broadly reactive reverse transcription-PCR assays to detect novel paramyxoviruses. J Clin Microbiol 46: 2652–2658. 18579717 10.1128/JCM.00192-08 PMC2519498

[19] Bracht AJ, Brudek RL, Ewing RY, Manire CA, Burek KA, et al (2006) Genetic identification of novel poxviruses of cetaceans and pinnipeds. Arch Virol 151: 423–438. 16328132 10.1007/s00705-005-0679-6

[20] Wellehan JF, Johnson AJ, Harrach B, Benko M, Pessier AP, et al (2004) Detection and analysis of six lizard adenoviruses by consensus primer PCR provides further evidence of a reptilian origin for the atadenoviruses. J Virol 78: 13366–13369. 15542689 10.1128/JVI.78.23.13366-13369.2004 PMC525023

[21] Reid SM, Ansell DM, Ferris NP, Hutchings GH, Knowles NJ, et al (1999) Development of a reverse transcription polymerase chain reaction procedure for the detection of marine caliciviruses with potential application for nucleotide sequencing. J Virol Methods 82: 99–107. 10507417 10.1016/s0166-0934(99)00088-9

